# The correlation between post-operative fentanyl requirements and *μ*-opioid receptor gene A118G polymorphism in patients undergoing radical gastrectomy

**DOI:** 10.3892/etm.2013.955

**Published:** 2013-02-11

**Authors:** FAN ZHANG, QIN LIAO, LI LI, SAI-YING WANG, RONG HU, YONG-ZHONG TANG, WEN OUYANG

**Affiliations:** Department of Anesthesiology, The Third Xiangya Hospital of Central South University, Changsha 410013, P.R. China

**Keywords:** fentanyl, analgesia, mu opioid receptors, single nucleotide polymorphism

## Abstract

The aim of this study was to investigate the effect of the *μ*-opioid receptor gene (OPRM1) A118G polymorphism on the requirement for post-operative fentanyl analgesia in patients undergoing radical gastrectomy. One hundred and twenty-eight gastric cancer patients scheduled to undergo radical gastrectomy under general anesthesia were enrolled in the study. Post-operative, patient-controlled intravenous analgesia of fentanyl was provided for satisfactory analgesia until 48 h after surgery. OPRM1 A118G was screened by DNA sequence analysis of polymerase chain reaction (PCR)-amplified DNA. Differences in fentanyl consumption and adverse effects were compared among the different genotypes at 24 and 48 h after surgery. The ranges of fentanyl dose in the 128 patients at 24 and 48 h after surgery were 5.4–17.3 *μ*g/kg and 12.4–29.9 *μ*g/kg, respectively. Among these patients, there were 54 wild-type homozygotes (AA), 53 heterozygotes (AG) and 21 mutant homozygotes (GG). The frequency of the G allele was 0.371 in the OPRM1 polymorphism. There were no significant differences in fentanyl dose or adverse effects, including nausea, vomiting and dizziness, for the OPRM1 A118G polymorphism (P>0.05). The OPRM1 A118G polymorphism does not play a significant role in post-operative fentanyl analgesic dose or post-operative nausea, vomiting and dizziness in patients undergoing radical gastrectomy.

## Introduction

Opioids, including morphine and fentanyl, have been widely used in clinical anesthesia and pain treatment for a number of years. However, the analgesic efficacy of opioids varies widely among individuals (1). Although several factors, including gender, age, weight, organ function and disease severity lead to differences in individual response to drugs, a number of studies have suggested that the inter-individual variability in drug dose may be mainly due to genetic factors (2–4).

The human mu-opioid receptor gene (OPRM1) encodes the *μ*-opioid receptor protein, which is the main target of the analgesic, fentanyl. Numerous single nucleotide polymorphisms (SNPs) have been identified in the OPRM1 gene [http://www.ncbi.nlm.nih.gov/SNP/snp_ref.cgi?locusId=4988]. The OPRM1 A118G mutation is the most common functional variation at position 118 in exon 1. This mutation leads to the conversion of the amino acid asparagine to aspartate at position 40 (N40D) on the extracellular N-terminal domain, which affects a putative glycosylation site of the receptor (5). Results from studies in patients with cancer pain or post-operative pain suggest that the A118G SNP has significant associations with morphine or alfentanil requirements for analgesia (6–10).

Fentanyl is one of the most commonly used opioid analgesics in acute and chronic pain therapy; however, there are few relevant published studies on its use. Several authors identified that subjects with the G allele of the OPRM1 A118G SNP were less sensitive to fentanyl or the fentanyl dose was greater compared to subjects with the AA allele, with regard to post-operative pain management or labor analgesia (11–14). However, other studies failed to identify such associations (15,16) or they obtained an opposing result (17). Similar to analgesic efficacy, the protection from analgesic opioid side-effects in G-allele carriers remains controversial (6,7,11). To further elucidate how the genetic variability in the OPRM1 gene contributes to opioid efficacy and side-effects, further studies are required in different races or ethnicities and in different types of pain or surgery with sufficient sample sizes (18).

Patients with acute post-operative pain following standardized surgical procedures may be optimal subjects for investigating such associations (1). Deriving gene-opioid associations from data sampled from patients with cancer pain is difficult since the mechanism, severity and nature of cancer pain differs substantially from patient to patient (10). Hence, in the present study, we selected Chinese Han patients undergoing radical gastrectomy to evaluate whether the genetic polymorphisms of OPRM1 affect post-operative fentanyl requirements for analgesia.

## Patients and methods

### Patients

This study was approved by the Institutional Ethics Committee of the Third Xiangya Hospital of Central South University, China. Signed, informed consent was obtained from all patients. From May 2008 to March 2010, patients classified as American Society of Anesthesiologists (ASA) physical status I-III, aged 20–75 years and undergoing radical gastrectomy were recruited into this study. Liver and renal functions of all patients were normal. Patients had no history of chronic pain, no long-term application of analgesics or cortisol-type drugs, no history of alcohol or drug abuse and no history of allergy to fentanyl. Exclusion criteria were a history of significant cardiovascular disease, diabetes mellitus or psychiatric disorders.

### Pre-operative psychological evaluation

Each patient received an evaluation by two psychiatrists of pre-operative anxiety and depression, as measured by the State-Trait Anxiety Inventory (STAI) and self-rating depression scale (SDS) on the night before surgery. STAI is a 40-item self-report measure of enduring (trait) and transient (state) anxiety symptoms (19). SDS is a 20-item self-administered scale designed to measure depression (20). STAI state and trait and SDS scores range from 20–80, with higher scores indicating the presence of state and trait anxiety or depression.

### Anesthetic methods

No pre-medication was used. All patients received general anesthesia, which was induced with 0.05 mg/kg midazolam, 0.2 mg/kg etomidate, 5 *μ*g/kg fentanyl and 0.12 mg/kg vecuronium. Following intubation of the trachea, the patients’ lungs were mechanically ventilated to maintain the end-tidal carbon dioxide partial pressure at 35–45 mmHg. Propofol and remifentanil were infused through micropumps and sevoflurane was inhaled for maintenance of anesthesia. Drug doses were adjusted according to changes in the auditory evoked potential and hemodynamics. An intermittent dose of vecuronium (0.03–0.06 mg/kg) was administered to maintain adequate surgical muscle relaxation. During surgery, the fentanyl infusion was discontinued once the surgeons had opened the abdominal cavity and the total dose of fentanyl was maintained at <12 *μ*g/kg to avoid residual effects of post-operative fentanyl dose. Electrocardiogram, noninvasive measurement of blood pressure, pulse oximetry and arterial blood gas measurements were monitored.

### Post-operative analgesia

Following surgery, all patients were sent to the post-anesthesia care unit (PACU). Once the trachea was extubated, the nurses in the PACU assessed pain severity. If the patients felt severe pain, they were intravenously injected with a 20 *μ*g bolus of fentanyl until a visual analog score (VAS; 0, no pain; 100, unbearable pain) <30 was attained. If they felt no pain, patient-controlled intravenous (i.v.) analgesia (PCIA) commenced if the patients perceived slight pain (VAS, 10–30). Patients were excluded if they received an opioid receptor antagonist. The electronic PCIA pump was filled with 30 *μ*g/kg fentanyl in 0.9% normal saline diluted to 240 ml. It was programmed to administer 1.5 ml/h background infusion with a 20 *μ*g bolus of fentanyl solution, with a 5 min lockout time. PCIA was continued for 48 h after surgery. Post-operative pain was controlled so that VAS was <30 at rest. When patients felt VAS >30 despite being on the PCIA pump, patients were able to increase the dose of fentanyl by pushing the bolus button. No rescue drugs other than i.v. fentanyl were used within the 48 h post-operative period. The total number of fentanyl boluses required during the PCIA coverage was also recorded. Due to the high incidence of nausea and vomiting, all patients received 8 mg ondansetron intravenously to prevent nausea and/or vomiting following surgery. Post-operative blood pressure, heart rate, pulse oxygen saturation and VAS were documented at 2, 6, 12, 24 and 48 h after surgery. The total amount of PCIA fentanyl was recorded at 24 and 48 h after surgery. Adverse effects associated with fentanyl administration, including nausea, vomiting and dizziness were assessed as events for the first post-operative 48 h.

### Genotyping assays

Peripheral venous blood (2 ml) from each patient was placed in an ethylenediamine tetraacetic acid (EDTA) tube. DNA was extracted from leukocytes using a standard phenol-chloroform procedure and was stored at 4–8°C. OPRM1 A118G genotyping was performed by DNA sequence analysis of polymerase chain reaction (PCR)-amplified DNA. Primers were designed by the primer design software Oligo 6.0 to cover the polymorphic site. OPRM1 A118G was amplified by PCR with the following primers: forward, GAAAAGTCTCGGTGCTCCTG and reverse, GGAGTAGAGGGCCATGATCG. The DNA sequence of the fragment was determined using an automated sequencer (ABI-PRISM 3730 Genetic Analyzer; Sangon Biotech Co. Ltd., Shanghai, China).

### Statistical analysis

The investigator conducting the clinical study and the laboratory personnel responsible for geno-typing were blinded to the clinical outcomes of the patients. Statistical analysis was performed using the SPSS v.18.0 software package (SPSS Inc., Chicago, IL, USA). P<0.05 was considered to indicate a statistically significant difference. Demographic and clinical data were presented as means ± standard deviation (SD), median and interquartile range or counts, as appropriate. The Chi-square test was used to detect Hardy-Weinberg equilibrium. According to the types of data, t-test, Fisher’s exact t-test or Mann-Whitney U test were used to evaluate whether the clinical parameters, including age, gender, body weight, height, duration of surgery and length of incision, exhibited significant differences among the different genotypes. In the present study, postoperative fentanyl doses at 24 and 48 h were not normally distributed. Therefore, nonparametric analyses, including the Mann-Whitney U test and Kruskal Wallis test were used to detect the effect of genotype on PCIA fentanyl consumption. The incidence of any adverse effects was analyzed using the Chi-square or Fisher’s exact t-test.

## Results

### General patient information

A total of 158 patients were enrolled in this study. Of these patients, 30 were not included in the study due to no available blood sample (n=6), genotype, analgesic pump failure (n=6), changes in condition (n=5), severe dizziness or vomiting (n=3), or withdrawal from the study (n=10). In the end, 128 patients completed the study. All patients were from a Han Chinese population. The clinical data of patients are summarized in Table I and no significant differences were detected among the genotype groups (P>0.05).

### Genotype and allele frequency of the OPRM1 A118G polymorphism

Among the 128 patients, there were 54 wild-type homozygotes (AA), 53 heterozygotes (AG) and 21 mutant homozygotes (GG). The frequency of the G allele was 0.371 for the OPRM1 polymorphism. The allele frequency was in Hardy-Weinberg equilibrium (P>0.05; Table II).

### Correlation between the OPRM1 A118G polymorphism and post-operative dose of fentanyl analgesic

The fentanyl doses for the 128 patients were 5.4–17.3 *μ*g/kg and 12.4–29.9 *μ*g/kg at 24 and 48 h after surgery, respectively. There were no clear correlations of fentanyl analgesic dose at 24 and 48 h after surgery among the genotype groups of OPRM1 A118G (P>0.05; Table I). At 48 h after surgery, the main side-effects of fentanyl were nausea, vomiting and dizziness (Table III). The incidences of post-operative nausea, vomiting and dizziness were 36.7% (47/128), 21.1% (27/128) and 33.6% (43/128) for patients with the AA, AG and GG genotypes, respectively. There were no significant differences in the incidence of nausea, vomiting or dizziness among the different geno-type groups (P=0.362, P=0.307 and P=0.233, respectively; Table III). Only two of the 128 patients (1.6%) developed mild pruritus. None of the patients developed respiratory depression or other serious side-effects.

## Discussion

In this study, the ranges of fentanyl dose among 128 patients were 5.4–17.3 and 12.4–29.9 *μ*g/kg for the first 24 and 48 h after surgery, respectively, which demonstrates that postoperative fentanyl analgesic dose varied significantly among individuals. A number of reasons, including the characteristics and intensity of external pain stimuli, age, gender, weight and pre-operative psychological state, may affect the perception of pain and the requirement for analgesics. We selected patients undergoing open radical gastrectomy, which is a surgical procedure that causes considerable post-operative pain, to identify differences in fentanyl dose. Surgery was performed on all patients by the same two surgeons. There were no differences in the time of surgery, the length of incision or patient age, gender, pre-operative psychological state, height or weight among the different genotype groups. Since the analgesic effect of fentanyl that was administered intra-operatively may outlast the duration of surgery and thus affect post-operative fentanyl use, particularly in patients who received large doses of fentanyl intra-operatively, we discontinued the infusion of fentanyl once the surgeons had opened the abdominal cavity and controlled the total dose of fentanyl so that it was <12 *μ*g/kg. Following surgery, all patients were administered with PCIA so they were in the same pain state (VAS=10–30). This gave all patients a true baseline pain intensity at the start of the observation period and the same endpoint (VAS=10–30). Doses of fentanyl administered post-operatively were also normalized to body weight. As described above, a number of factors affect post-operative fentanyl dose, so our intent was to be as consistent as possible across patients. When we ruled out interference from these confounding factors, we studied the association between the OPRM1 A118G polymorphism and post-operative dose of fentanyl in Han Chinese patients undergoing radical gastrectomy.

We did not find a clear correlation between the OPRM1 A118G SNP and fentanyl analgesic dose at 24 or 48 h after surgery. Results similar to ours have been reported for labor analgesia and cancer pain (15,16). Wong *et al* (15) identified no difference in the median duration of intrathecal use of fentanyl in labor analgesia among patients with the OPRM1 A118G polymorphism. Klepstad *et al* (16) reported that OPRM1 A118G demonstrates no significant association with fentanyl dose in a European genetic association study of 394 cancer pain patients. Our findings were, however, contrary to those of a number of other studies (11–14). These studies reported that fentanyl is less effective in subjects with the G allele of the OPRM1 A118G SNP than in those with the A allele and subjects with the G allele required more fentanyl for adequate post-operative pain control than those with the A allele. Kasai (18) considered that these controversial results may be attributable to the different minor allele frequencies between races and ethnicities in the sample populations. In the above mentioned four studies, however, the samples were all from Asian individuals. The frequencies of the mutant G allele were 45 and 43.8% in two Japanese populations (11,13) and 31.3 and 37.1% in a Chinese population in a previous study (10) and in the present study, respectively. Use of the same population with such high mutation frequencies may have enabled us to conduct a more reliable statistical analysis and make a better comparison. These studies and our study were all related to acute post-operative pain.

There are two possible reasons for the conflicting findings. First, the analgesia method and analgesic used for post-operative pain differed. In the present study, we administered only fentanyl by PCIA with no other rescue analgesic. In contrast, in the Hayashida *et al* (14) study, 138 patients that underwent major, open abdominal surgery received primarily continuous epidural analgesia with fentanyl or morphine, with a rescue analgesic, including buprenorphine, pentazocine and pethidine and non-steroidal anti-inflammatory drugs (NSAIDs). Analgesic requirements in the first 24 h post-operative period were determined as the sum of systemic fentanyl equivalent doses of all opioids and NSAIDs used for analgesia during the first 24 h after surgery. We consider that the conversion may not reflect precise fentanyl doses due to the varying properties and various routes of administration for different analgesics. Secondly, in the Fukuda *et al* (12) study, although the analgesic effects of fentanyl evaluated with the cold pressor prior to surgery were reduced in subjects carrying the G allele compared with subjects not carrying this allele, the A118G SNP demonstrated no significant association with 24 h post-operative fentanyl use, peri-operative fentanyl use, total peri-operative analgesic use or VAS at 3 or 24 h in patients undergoing orofacial cosmetic surgery. Thus, this result was consistent with ours with regard to the association between OPRM1 A118G polymorphism and post-operative dose of fentanyl.

As with clinical studies, not all data from *in vitro* studies of the OPRM1 A118G mutation and opioid interaction supported positive results. Bond *et al* (21) identified that the mutant *μ*-opioid receptor did not show altered binding affinities for the majority of opioid peptides and alkaloids, including fentanyl, although the variant receptor expressed in AV-12 cells had a three-fold higher binding affinity for *β*-endorphin than the wild-type opioid receptor. Two other studies also reported no difference in the binding characteristics of ligands to the mutant *μ*-opioid receptor (22,23). These results suggest that the A118G polymorphism does not change the overall binding properties of the *μ*-opioid receptor. In a study by Mahmoud *et al* (24) in sensory neurons isolated from a humanized mouse model, the potency and efficacy of fentanyl were similar for 118 AA and 118 GG sensory neurons and the fentanyl pharmacological profile for the two groups of neurons was also similar, which was consistent with our results. Unlike the effects of fentanyl, morphine was less potent and efficacious in 118 AA neurons and morphine-mediated analgesia in 118 GG mice was significantly reduced compared with the 118 AA mice, also supporting the previous clinical association studies on morphine and the OPRM1 A118G polymorphism. It is unclear why fentanyl and morphine exert a different pharmacological profile in the mouse model. It may be due to opioid-specific responses, so the results from one drug may not easily extrapolate to other drugs.

Post-operative nausea and vomiting are common side-effects of fentanyl. In the present study, the incidences of post-operative nausea and vomiting were 36.7% (47/128) and 21.1% (27/128), respectively, for all the patients, which were much higher than the previously reported range of 5.6–28.5% (6,7,10,25,26). Patients undergoing radical gastrectomy are particularly prone to post-operative nausea and vomiting due to the site of surgery and stimulation of the gastric tube. However, we identified that there was no significant difference in nausea and vomiting among the different genotype groups. Our results were similar to previous reports (6,26). A number of factors are known to affect post-operative nausea and vomiting, including age, gender, type of surgery and anesthesia, pre-operative psychological state and the particular drugs used following surgery. In this study, there were no significant differences in the above factors, including opioid dose, among the genotype groups. In contrast, Tan *et al* (7), Klepstad *et al* (10) and Sia *et al* (25) all reported that the AA group had the highest nausea score and incidence compared with the other two groups. However, the above two studies (10,25) also showed that the nature of pain was malignant pain and post-cesarean pain. These patients may have different physiological or pathological conditions, including increased hormone levels in pregnant women. Another difference was the use of morphine, which has different pharmacological features to fentanyl, despite belonging to the same class of drug. Fentanyl has inhibitory effects on gastrointestinal motility, which is thought to be the main reason for frequent nausea and vomiting. Walldén *et al* (27) identified a large variation in gastric myoelectrical activity following intravenous fentanyl administration; however, this was not explained by the OPRM1 A118G polymorphism. Therefore, we consider that there may be no correlation between the nausea and vomiting induced by fentanyl and the OPRM1 A118G genotypes.

Post-operative dizziness is often observed during PCIA by fentanyl. In this study, the incidence of dizziness was 33.6% (43/128), similar to the report by Lin *et al* (28) and it mainly occurred in the first 24 h after surgery. Similarly, we did not observe an association between the OPRM1 A118G polymorphism and the incidence of dizziness. However, the adverse effects were assessed as events, not on a scale, which may not reflect the real changes and may have led to differences in the results.

In the present study, we controlled a number of confounding factors, including biological and psychological variation, and analgesia management had the same start- and endpoint with intravenous fentanyl, which reflects the real gene-dose association. However, our sample size may be inadequate to make comments on negative genetic effects on fentanyl dose and side-effects. Additionally, we studied only one polymorphism of the OPRM1 gene and other genetic variations in OPRM1 and other candidate genes may also play a role in the response to opioids. Moreover, the mixed-gender study population may have increased variability in post-operative fentanyl requirement, although there was no statistical difference in gender between the different genotypes.

Multiple, complex biological systems are involved in opioid pharmacology and pain processing. Age, gender, body weight, psychological state, disease conditions, tolerance to side-effects, genetic variation, race and ethnicity cause different opioid requirements for patients. For different drugs, there are various routes of administration, potential interference from drug interactions and different pharmacokinetics and pharmacodynamics. The differences in the nature and intensity of nociceptive stimuli, including labor pain, acute post-operative pain and chronic pain, as well as the modalities used to describe pain perception and analgesic responses, may also contribute to inconsistent findings. In addition, although a number of functional analyses of the A118G SNP have been performed, the results of these studies are also controversial in animal and clinical studies. Therefore, further studies are required to clarify the association between the A118G SNP and treatment efficacy or side-effects of fentanyl with sufficient samples for each effect size in different races and ethnicities.

In conclusion, our study suggests that the OPRM1 A118G polymorphism does not play a significant role in either post-operative fentanyl analgesic dose or the incidence of postoperative side-effects in Han Chinese patients undergoing radical gastrectomy.

## Figures and Tables

**Table I:** Demographic and clinical data for genotyped groups.

Parameters	AA	AG	GG	P-value
N	54	53	21	
Age (years)	50.7±12.4	53.8±12.8	54.1±11.9	0.363
Male/female	30/24	32/21	14/7	0.666
Body weight (kg)	54.5±10.5	54.9±10.0	57.0±10.6	0.617
Height (cm)	161.5±7.9	161.4±7.2	163.6±7.1	0.480
Duration of surgery (min)	206.8±56.2	193.9±71.8	182.9±70.8	0.322
Length of incision (cm)	19.0±2.6	19.8±3.7	19.3±4.7	0.473
Pre-operative state anxiety	40.3±8.1	40.7±9.0	38.5±8.3	0.719
Pre-operative trait anxiety	41.8±9.0	43.4±10.6	44.8±6.6	0.577
Pre-operative depression	38.4±8.7	34.8±6.0	35.7±6.6	0.165
24 h post-operative fentanyl dose (*μ*g/kg)	10.2 (7.2–15.7)	8.5 (7.0–13.3)	10.2 (7.8–11.7)	0.679
48 h post-operative fentanyl dose (*μ*g/kg)	19.2 (15.0–26.3)	18.3 (14.3–24.1)	19.6 (14.0–25.8)	0.810
VAS pain score at 24 h (mm)	19.0±4.0	19.5±4.1	18.4±3.8	0.572
VAS pain score at 48 h (mm)	18.6±3.3	19.2±3.5	18.6±3.5	0.639

Data are expressed as mean ± standard deviation or median (interquartile range). AA, wild-type homozygotes; AG, heterozygotes; GG, mutant homozygotes.

**Table II:** OPRM1 A118G genotype and allele frequency distribution.

OPRM1 A118G genotypes				OPRM1 A118G alleles		
	
AA	AG	GG	Total	A	G	Total
54	53	21	128	161	95	256
(42.2%)	(41.4%)	(16.4%)		(62.9%)	(37.1%)	

χ^2^=1.632, P>0.05. AA, wild-type homozygotes; AG, heterozygotes; GG, mutant homozygotes.

**Table III:** Incidence of nausea, vomiting and dizziness among the genotyped groups of the OPRM1 A118G polymorphism.

	AA	AG	GG	*χ^2^*	P-value
N	54	53	21		
Nausea					
No	38 (70.4)	31 (58.5)	12 (57.1)		
Yes	16 (29.6)	22 (41.5)	9 (42.9)	2.032	0.362
Vomiting					
No	46 (85.2)	40 (75.5)	15 (71.4)		
Yes	8 (14.8)	13 (24.5)	6 (28.6)	2.360	0.307
Dizziness					
No	40 (74.1)	31 (58.5)	14 (66.7)		
Yes	14 (25.9)	22 (41.5)	7 (33.3)	2.912	0.233

Data are presented as N (%). AA, wild-type homozygotes; AG, heterozygotes; GG, mutant homozygotes.
